# Using antidepressants and the risk of stroke recurrence: report from a national representative cohort study

**DOI:** 10.1186/s12883-015-0345-x

**Published:** 2015-06-05

**Authors:** Hsiao-Ting Juang, Pei-Chun Chen, Kuo-Liong Chien

**Affiliations:** Institute of Epidemiology and Preventive Medicine, College of Public Health, National Taiwan University, Taipei, Taiwan; Biostatistical Center for Clinical Research, Chang Gung Memorial Hospital, Taoyuan, Taiwan; Clinical Informatics and Medical Statistics Research Center, Chang Gung University College of Medicine, Taoyuan, Taiwan

## Abstract

**Background:**

Evidence about the association between antidepressants and the risk of stroke recurrence was scanty. This study evaluated the risk of stroke recurrence according to using antidepressants in patients with stroke from a national representative cohort.

**Methods:**

This cohort study followed 16770 patients aged > =20 years who had an incident stroke from 2000 to 2009 from the National Health Insurance Research Database in Taiwan. Records of each antidepressant prescription were obtained during follow-up. The types of antidepressants were categorized by Anatomical Therapeutic Chemical classification system: tricyclic antidepressants (TCAs), selective serotonin reuptake inhibitors (SSRIs), monoamine oxidase inhibitors (MAOIs), and other antidepressants. The main outcome was a recurrent stroke during the follow-up period. The time-dependent Cox proportional hazards model was used in the analyses.

**Results:**

During 63715 person-years of follow-up, we documented 3769 events for stroke recurrence. Antidepressants use was associated with an increased risk of stroke recurrence (adjusted hazard ratio [HR], 1.42; 95 % confidence interval [C.I.], 1.24–1.62), especially for ischemic stroke (HR, 1.48; 95 % C.I., 1.28–1.70), but not for hemorrhagic stroke (HR, 1.22; 95 % C.I., 0.86–1.73). The increased risk of stoke recurrence was found for TCAs use only (HR, 1.41; 95 % C.I., 1.14–1.74), SSRIs use only (HR, 1.31; 95 % C.I.,1.00–1.73),use of other types of antidepressants only(HR, 1.46; 95 % C.I.,1.15–1.84), or use of multiple types of antidepressants (HR, 1.84; 95 % C.I.,1.04–3.25).

**Conclusions:**

We demonstrated that use of antidepressants was associated with an increased risk of stroke recurrence, especially in ischemic stroke among Taiwanese. Further studies are warranted to confirm the possible underlying mechanisms of these findings.

## Background

Stroke is one of the leading causes of adult disability and mortality worldwide, resulting in tremendous socioeconomic burden [1, 2]. The recurrence rate of stroke readmission within one year was 13 % in Taiwan [3]. Compared with incident stroke events, recurrent events were likely to have higher mortality rates, greater levels of disability, and increased costs [4].

Use of antidepressants had positive effect on the management of stroke patients due to the reduction in incidence rate of post-stroke depression [5] and improvement in functional recovery [6, 7], but use of antidepressants also increased side effects [8]. In the cochrane review, the authors concluded that SSRIs might improve recovery after stroke, and that there was heterogeneity between published trials and methodological limitations [9]. Recent epidemiological studies had shown antidepressants use was associated with an increased risk of developing stroke [10]; however, data on stroke recurrence were limited [11, 12]. Moreover, some studies have indicated depression was associated with a higher risk of stroke [13], including recurrent events [11]. The association of fatal stroke in patients with depression who receiving antidepressants was even stronger [14]. The role of depression in the association between antidepressants use and stroke recurrence remains unclear. We used the National Health Insurance Research Database in Taiwan to evaluate whether antidepressants use is associated with increased risk of stroke recurrence.

## Methods

### Data source and study subjects

This cohort study used the Longitudinal Health Insurance Database (LHID), a sub-dataset of National Health Insurance (NHI) Research Database containing healthcare claims between 1996 and 2010 for a cohort of one million people randomly sampled from beneficiaries of NHI. The NHI provides coverage to 99 % or more of Taiwanese population. LHID consists of many data files, including inpatient records, ambulatory care records, contracted pharmacies records, and registries for beneficiaries and contracted medical facilities.

**Fig. 1:**
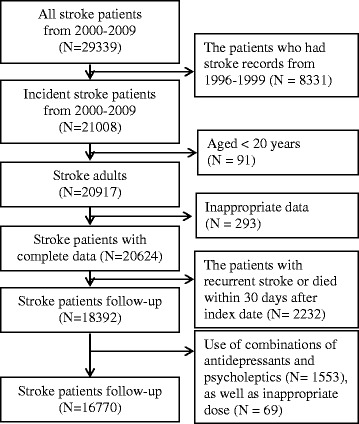
Patients Inclusion Chart

Included in this study were patients had a first hospitalization with diagnosis of stroke during 2000 and 2009. The date of the first hospitalization for stroke was identified as the index date. Stroke was identified by principal diagnosis with ICD-9-CM code (International Classification of Diseases, 9th revision, Clinical Modification codes) 430 to 432 for hemorrhagic stroke and 433 to 437 for ischemic stroke. Those who had any diagnosis of stroke from 1996 to 1999 were excluded to reduce the possibility of including prevalent stroke cases. We further excluded patients who were aged <20 years (N = 91), who had inappropriate data with index date after the date of withdrawing from insurance (N = 293), and patients with recurrent stroke or died within 30 days after index date (N = 2232). Patients who had use of combinations of antidepressants and psycholeptics (amitriptyline-psycholeptics or melitracen-psycholeptics) (N = 1553) or too high dose (>3 DDDs, defined daily doses) (N = 69) during the follow-up period were also excluded. Therefore, the study included 16770 patients with stroke (Fig. 1). This study was approved by the institutional ethics review board at the National Taiwan University Hospital.

### Exposure to antidepressants and covariates

For each patient, the records (detail information of drug code by Bureau of National Health Insurance, total dosage and days of use for each prescription) of prescriptions of antidepressants were obtained during the follow-up. The types of antidepressants were categorized by Anatomical Therapeutic Chemical (ATC) classification system [15]: non-selective monoamine reuptake inhibitors (tricyclic antidepressants, TCAs), selective serotonin reuptake inhibitors (SSRIs), monoamine oxidase inhibitors (MAOIs), and other antidepressants. The average dosage for the each prescription of antidepressants per day was calculated. We classified average dose by defined daily doses (DDDs) [15], as defined by the World Health Organization, into <0.5, 0.5–1, and > =1 DDDs.

Other covariates included sex, age, related-disease and prescriptions of other drugs within one year before the index date such as antipsychotics (ATC code: N05A), antithrombotic agents included anticoagulant (ATC code: B01AA03) and antiplatelet (ATC code: B01AC06, B01AC04, B01AC05, B01AC23, B01AC07), anti-inflammatory (ATC code: M01A), antidepressants use before stroke (ATC code: N06A), depression (ICD-9-CM code: 296.2, 296.3, 300.4, 311), other mood disorders or (ICD-9-CM code: 296.0, 296.1, 296.4–296.9) anxiety (ICD-9-CM code: 300.0), atrial fibrillation (ICD-9-CM code: 427.3), coronary heart disease (ICD-9-CM code: 410–414), congestive heart failure (ICD-9-CM code: 428), chronic obstructive pulmonary disease (ICD-9-CM code: 490,491,492,493,494,495,496), cancer (ICD-9-CM code: 140–208), diabetes mellitus or medicine treatment (ICD-9-CM code: 250; ATC code: A10), hypertension or medicine treatment (ICD-9-CM code: 401–405; ATC code: C02, C03, C07, C08, C09), and hyperlipidemia or medicine treatment (ICD-9-CM code: 272; ATC code: C10). These variables were all possible confounders between antidepressants and stroke recurrence. The presence of depression during the follow-up was also obtained.

### Main outcome

The main outcome was a re-hospitalization with hemorrhagic stroke or ischemic stroke during the follow-up period. The follow-up started from the index date, which was between 2000 and 2009, and ended on the date of stroke recurrence, the date of withdrawing from insurance, or date of termination of this study, December 31, 2010; whichever came first.

### Statistical analyses

Demographic and clinical characteristics between use of antidepressants group (at least one prescription of antidepressants during follow-up) and non-use were compared by the chi-square test. We also divided the type of antidepressants into TCAs, SSRIs, MAOIs, others, or multiple types, as well as dose groups into >0.5, 0.5–1, and > =1 DDDs to perform the descriptive analyses. Because of the time-varying nature of drug use, we defined the duration of antidepressants exposure as days of use for each prescription from database at ambulatory care, and contracted pharmacies, but not at inpatient records because data on days supplied for each prescription were not obtained. We classified the exposure status into “use” during the duration of antidepressants and “non-use” during the days of no prescriptions. We computed their incidence rate (per 1000 person-year) by dividing number of events of recurrent stroke with person-years of exposure to each antidepressant. We used the Simon and Makuchmethod [16] to graphically represent survival curves for time to use of antidepressants by Stata to compute ‘Kaplan-Meier’ estimates for time-dependent covariate**s** [17].

We used univariable and multivariable models to estimate the hazard ratios (HR) and 95 % confidence interval (C.I.) by the Cox proportional hazards model with time-varying antidepressants use to assess the association between each antidepressants use category and recurrent stroke with “non-use” as the reference group and adjusted the models for demographic and clinical characteristics. Moreover, subgroup analyses were performed including antidepressants use before stroke or not (prevalent users, or new users), depression status (no-diagnosed depression, prevalent depression, or post-stroke depression with newly-diagnosed depression), other drugs use, and other disease. Finally, we performed two sensitivity analyses. First, the duration of antidepressants exposure was redefined by adding seven days to the end of days supply for each prescription in order to take into account for the potential carry over effect and gaps in therapies [10]. Second, we added the prescriptions of antidepressants in the inpatient settings. Because the inpatients claims do not contain information on the days supply for medications, we defined the duration of antidepressants exposure using the length of hospital stay. All analyses were carried out with SAS 9.2 and Stata 10. A two-sided p value < 0.05 was considered statistically significant.

## Results

Of the 16770 stroke patients with mean age of 67.3 years, 4695 (28 %) were taking any antidepressant during follow-up (median, 3.04 years; interquartile range, 1.31–5.64). Table 1 showed the baseline characteristics of subjects with use of antidepressants compared with those with non-use of antidepressants. Stroke patients with use of antidepressants were more female and ischemic stroke, as well as had a higher prevalence of diabetes mellitus, hyperlipidemia, chronic obstructive pulmonary disease, cancer, depression, anti-inflammatory, antithrombotic agents, antipsychotics, and antidepressants use before stroke. Among4695stroke patients with antidepressants use, the most commonly type of antidepressants was TCAs use only (n = 1814 [38 %]) and SSRIs use only (n =661 [14 %]), and MAOIs use only (n = 95 [2 %]) were less common. In dose groups, the group of <0.5 DDDs (50 %) was common, and the proportion of the group of 0.5–1 DDDs and •1 DDDs were 21 % and 28 % respectively.

**Table 1:** Characteristics of Study Subjects

	Use of Antidepressants		Non-use of Antidepressants		
	(N = 4695, 28 %)		(N = 12075, 72 %)		
	n	%	n	%	*p*-value
**Sex (Female)**	2046	(43.6)	4806	(39.8)	<.001
**Age (years)**					
18–44	240	(5.11)	797	(6.60)	<.001
45–54	593	(12.6)	1580	(13.1)	<.001
55–64	957	(20.4)	2321	(19.2)	<.001
64–74	1536	(32.7)	3399	(28.1)	<.001
> = 75	1369	(29.2)	3978	(32.9)	<.001
**Stroke type**					
Hemorrhagic stroke	761	(16.2)	2493	(20.6)	<.001
Ischemic stroke	3934	(83.8)	9582	(79.4)	<.001
**Antipsychotics**	1515	(32.3)	3338	(27.6)	<.001
**Antithrombotic agents**					
anticoagulant	133	(2.83)	395	(3.27)	0.14
antiplatelet	3130	(66.7)	7240	(60.0)	<.001
**Anti-inflammatory**	3970	(84.6)	9354	(77.5)	<.001
**Antidepressants use before index date**	1617	(34.4)	964	(7.98)	<.001
**Depression**	397	(8.46)	176	(1.46)	<.001
**Anxiety or other mood disorders** ^**a**^	354	(7.54)	449	(3.72)	<.001
**Atrial fibrillation**	302	(6.43)	935	(7.74)	0.004
**Coronary heart disease**	1065	(22.7)	2534	(21.0)	0.016
**Congestive heart failure**	317	(6.75)	959	(7.94)	0.009
**Chronic obstructive pulmonary disease**	804	(17.1)	1912	(15.8)	0.042
**Cancer**	59	(1.26)	88	(0.73)	<.001
**Diabetes mellitus** ^Ϯ^	1775	(37.8)	4120	(34.1)	<.001
**Hypertension** ^Ϯ^	4210	(89.7)	10724	(88.8)	0.11
**Hyperlipidemia** ^Ϯ^	1673	(35.6)	3765	(31.2)	<.001

^a^Other mood disorders included manic disorder, bipolar disorder or anxiety

^Ϯ^A patient was identified as having these comorbidities if he or she had a ICD-9-CM diagnosis code of that comorbidity on outpatient or inpatient claims, or a prescription for its medications, i.e., antidiabetic medications for diabetes, antihypertensives for hypertension, and antihyperlipidemic agents for hyperlipidemia

**Fig. 2:**
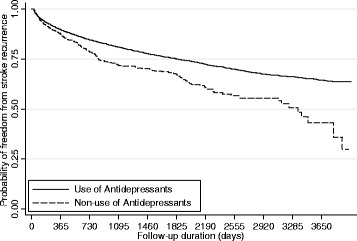
Survival Curves of Stroke Recurrence for Antidepressants use during All Follow-up time Using the Simon and Makuch Method

Figure 2 depicted increased risk of recurrent stroke with antidepressants use. In Cox proportional hazards model with time-varying antidepressants use for the univariate analyses, use of antidepressants was found to have a higher risk of stroke recurrence (HR,1.49;95 % confidence interval [C.I.],1.30–1.70) (Table 2). After controlling for potential confounding variables, use of antidepressants also had a significantly increased risk of stroke recurrence (HR, 1.42; 95 % C.I.,1.24–1.62). A significantly increased risk of stroke recurrence was found with TCAs use only (HR, 1.41; 95 % C.I.,1.14–1.74), SSRIs use only (HR, 1.31; 95 % C.I.,1.00–1.73), other antidepressants use only (HR, 1.46; 95 % C.I.,1.15–1.84), and multiple types use (HR, 1.84; 95 % C.I.,1.04–3.25). We had similar results for the increased risk of recurrent stroke with antidepressants use in different dose groups of <0.5 DDDs (HR, 1.40; 95 % C.I.,1.17–1.67), 0.5–1 DDDs (HR, 1.46; 95 % C.I.,1.10–1.94), and 1 DDDs (HR, 1.43; 95 % C.I.,1.11–1.84). Table 3 summarizes the association between subtype of stroke and antidepressants use category. We found a similar pattern in ischemic stroke recurrence and a non-significantly increased risk of hemorrhagic stroke recurrence.

**Table 2:** The Risk of Stroke Recurrence in association with Antidepressants

	N for Stroke Recurrence/Person-years	Rate per 1000 Person-Years	Crude HR	(95 % CI)	Adjusted HR^a^	(95 % CI)
Non-use of Antidepressants	3532/59400	59.5	1		1	
Use of Antidepressants	237/2440	97.1	1.49	(1.30, 1.70)	1.42	(1.24, 1.62)
Type of Antidepressants						
TCAs only	88/910	96.8	1.48	(1.20, 1.83)	1.41	(1.14, 1.74)
SSRIs only	53/617	86.0	1.38	(1.05, 1.81)	1.31	(1.00, 1.73)
MAOIs only	10/91	110.4	1.45	(0.78, 2.70)	1.41	(0.76, 2.63)
Others only	74/732	101.1	1.53	(1.22, 1.93)	1.46	(1.15, 1.84)
Multiple types	12/91	132.4	1.93	(1.09, 3.40)	1.84	(1.04, 3.25)
Dose Group^+^						
<0.5 DDDs	126/1294	97.4	1.47	(1.23, 1.75)	1.40	(1.17, 1.67)
0.5–1 DDDs	48/492	97.6	1.54	(1.16, 2.05)	1.46	(1.10, 1.94)
> = 1 DDDs	63/654	96.4	1.50	(1.17, 1.92)	1.43	(1.11, 1.84)

HR indicates hazard ratio; 95 % CI: 95 % confidence interval

^+^Dose Group = The Average Dose of the Prescriptions for Antidepressants per day/Defined Daily Dose; unit: DDDs

^a^Adjusted for sex, age group, stroke type of the first hospitalization, antipsychotics, antithrombotic agents, anticoagulant, antiplatelet, antidepressant drugs use before stroke, depression, anxiety or other mood disorders, atrial fibrillation, coronary heart disease, congestive heart failure, COPD, cancer, diabetes mellitus or medicine treatment, hypertension or medicine treatment, hyperlipidemia or medicine treatment

**Table 3:** The Risk of Recurrence of Ischemic Stroke and Hemorrphagic Stroke in association with Antidepressants

	Ischemic Stroke						Hemorrhagic Stroke					
	Number of Recurrent Stroke/Person-years	Rate per 1000 Person-Years	Crude HR	(95 % CI)	Adjusted HR^a^	(95 % CI)	Number of Recurrent Stroke/Person-years	Rate per 1000 Person-Years	Crude HR	(95 % CI)	Adjusted HR^a^	(95 % CI)
Non-use of Antidepressants	3012/61194	49.2	1		1		681/68749	9.9	1		1	
Use of Antidepressants	217/2521	86.1	1.58	(1.38, 1.82)	1.48	(1.28, 1.70)	35/3098	11.3	1.10	(0.78, 1.54)	1.22	(0.86, 1.73)
Type of Antidepressants												
TCAs only	83/930	945.2	1.64	(1.32, 2.04)	1.53	(1.23, 1.90)	12/1113	10.8	1.03	(0.58, 1.83)	1.08	(0.61, 1.91)
SSRIs only	49/653	649.7	1.46	(1.10, 1.93)	1.34	(1.01, 1.79)	8/794	10.1	1.03	(0.51, 2.06)	1.23	(0.61, 2.48)
MAOIs only	8/93	92.6	1.36	(0.68, 2.72)	1.24	(0.62, 2.49)	2/111	18.0	1.53	(0.38, 6.11)	2.15	(0.54, 8.68)
Others only	11/92	761.1	1.59	(1.24, 2.03)	1.50	(1.17, 1.92)	10/963	10.4	1.01	(0.54, 1.88)	1.12	(0.60, 2.10)
Multiple types	66/756	92.2	2.06	(1.14, 3.73)	1.95	(1.08, 3.54)	3/117	25.7	2.51	(0.81, 7.80)	2.78	(0.89, 8.68)
Dose Group^+^												
<0.5 DDDs	116/1322	87.7	1.58	(1.31, 1.90)	1.49	(1.23, 1.79)	16/1638	9.8	0.93	(0.57, 1.53)	1.00	(0.61, 1.64)
0.5–1 DDDs	46/502	91.7	1.73	(1.29, 2.31)	1.59	(1.19, 2.13)	6/617	9.7	0.97	(0.44, 2.18)	1.11	(0.50, 2.49)
> = 1 DDDs	55/697	78.9	1.48	(1.13, 1.93)	1.37	(1.05, 1.80)	13/844	15.4	1.53	(0.89, 2.66)	1.81	(1.04, 3.16)

HR indicates hazard ratio; 95 % CI: 95 % confidence interval

^+^Dose Group = The Average Dose of the Prescriptions for Antidepressants per day/Defined Daily Dose; unit: DDDs

^a^Adjusted for sex, age group, stroke type of the first hospitalization, antipsychotics, antithrombotic agents, anticoagulant, antiplatelet, antidepressant drugs use before stroke, depression, anxiety or other mood disorders, atrial fibrillation, coronary heart disease, congestive heart failure, COPD, cancer, diabetes mellitus or medicine treatment, hypertension or medicine treatment, hyperlipidemia or medicine treatment

In subgroup analysis (Table 4), recurrent stroke risk estimates were similar among different depression statuses. The increased risk of recurrent stroke with antidepressants use in prevalent users (HR, 1.23; 95 % C.I.,0.99–1.53) was lower than in new users (HR, 1.57; 95 % C.I.,1.32–1.86). The data provided significant differences (p for interaction = 0.01) in risk between diabetes users (HR, 1.70; 95 % C.I.,1.41–2.05) and non-diabetes users (HR, 1.20; 95 % C.I.,0.99–1.45). The data did not show significant evidence of differences in risk related to other drugs use and other disease. In sensitivity analyses, we found that the stroke risk was slightly lower but still significant with antidepressants use in duration plus 7 days (HR, 1.33; 95 % C.I.,1.17–1.52) and adding inpatient records (HR, 1.19; 95 % C.I.,1.05–1.36).

**Table 4:** The Risk of Stroke Recurrence in association with Antidepressants in Subgroup of Other Drugs Use and Comorbidities

	Adjusted HR^a^	(95 % CI)
Without antipsychotics	1.40	(1.18, 1.66)
With antipsychotics	1.45	(1.16, 1.82)
Without anticoagulant	1.44	(1.25, 1.65)
With anticoagulant	1.24	(0.76, 2.00)
Without antiplatelet	1.41	(1.23, 1.62)
With antiplatelet	1.52	(0.72, 3.22)
Without anti-inflammatory	1.29	(0.90, 1.85)
With anti-inflammatory	1.44	(1.24, 1.67)
Without anxiety or other mood disorders	1.40	(1.07, 1.82)
With anxiety or other mood disorders	1.42	(1.21, 1.66)
Without atrial fibrillation	1.40	(1.22, 1.62)
With atrial fibrillation	1.57	(0.98, 2.50)
Without coronary heart disease	1.36	(1.16, 1.60)
With coronary heart disease	1.59	(1.24, 2.05)
Without congestive heart failure	1.44	(1.25, 1.65)
With congestive heart failure	1.15	(0.68, 1.97)
Without chronic obstructive pulmonary disease	1.49	(1.29, 1.73)
With chronic obstructive pulmonary disease	1.09	(0.77, 1.54)
Without cancer	1.42	(1.24, 1.63)
With cancer	0.94	(0.26, 3.32)
Without diabetes mellitus	1.20	(0.99, 1.45)
With diabetes mellitus^+^	1.70	(1.41, 2.05)
Without hypertension	1.25	(0.69, 2.27)
With hypertension	1.42	(1.24, 1.64)
Without hyperlipidemia	1.41	(1.18, 1.68)
With hyperlipidemia	1.42	(1.15, 1.76)
**Antidepressants use before index date**		
No--New Users	1.57	(1.32, 1.86)
Yes--Prevalent Users	1.23	(0.99, 1.53)
**Depression at Baseline (index date) or Post-stroke**		
No Depression	1.55	(1.32, 1.82)
Depression at Baseline	1.49	(0.99, 2.26)
Post-stroke Depression	1.44	(1.01, 2.07)

HR indicates hazard ratio; 95 % confidence interval

^+^p-value for the interaction term of diabetes was 0.01, and that of other variables were all >0.05

^a^Adjusted for sex, age group, stroke type of the first hospitalization, antipsychotics, antithrombotic agents, anticoagulant, antiplatelet, antidepressant drugs use before stroke, depression, anxiety or other mood disorders, atrial fibrillation, coronary heart disease, congestive heart failure, COPD, cancer, diabetes mellitus or medicine treatment, hypertension or medicine treatment, hyperlipidemia or medicine treatment

## Discussion

This study showed that stroke patients prescribed for antidepressants had 40 % greater risk of stroke recurrence. The association was stronger for ischemic stroke than for hemorrhagic stroke. The increased risk was observed in association with prescriptions for antidepressants subclass of TCAs, SSRIs, the group of others, and multiple types. In addition, a higher risk of stroke recurrence with antidepressants among diabetic patients was found.

Few epidemiological studies have reported the association between antidepressants use and risk of stroke recurrence. A study in China found an elevated risk of recurrent stroke after 1-year follow-up in antidepressants users, but the association was statistically non-significant, partly because of small sample size (RR, 1.96; 95 % C.I.,0.95–4.04) [11]. Another study in Denmark using propensity score–matching analysis focused on SSRI use [12]. SSRI exposure was associated with statistically non-significant lower risk of recurrence of ischemic stroke (RR, 0.67; 95 % C.I.,0.44–1.02), and non-significantly higher risk of intracranial bleedings (RR, 1.14; 95 % CI, 0.62–2.12) [12]. The discrepancy in findings among these studies and ours may reflect different methodology among studies, including differences in patient populations, reported type of stroke, and methods of data analysis. We used Cox models with antidepressant exposure as time-dependent covariates to take into account for switch between classes of antidepressants and use to non-use. The actual number of days prescribed was the days of exposure in our analysis. However, all drugs in the Denmark study were included as time-dependent variables with an assumed average prescription length of 90 days. The extent of drug exposure in their study might be different from that in ours. A cochrane review [9] has concluded that SSRIs might improve recovery after stroke. However, different from our study, the outcomes assessed in the review were neurological impairment or depression after stroke, but not stroke recurrence. Furthermore, the authors of the review found high heterogeneity between trials and methodology limitations in these trials. Well-designed trials are needed before a firm conclusion can be made.

The biological mechanisms have been proposed for the association between antidepressants and stroke. Antidepressant drugs inhibit the re-uptake receptors or the activity of metabolic enzymes, thereby increasing the concentration of serotonin, which induces local vasoconstriction and increases risk of ischemic stroke [18, 19]. In contrast, evidence also revealed that antidepressants may inhibit platelet activity and aggregation, thereby preventing ischemic events [20–22]. This mechanism also could explain the underlying association between antidepressants and the increased risk of bleeding. However, epidemiological studies showed inconclusive results in the association between antidepressants and risk of hemorrphagic stroke [23–26]. A study of meta-analysis reported increased risk of brain hemorrhage associated with SSRIs [23], whereas other studies, in line with our analysis, suggested no increased risk [24–26]. Of note, different from previous studies, our analysis focused on risk of recurrent stroke. More investigations with stroke recurrence as the primary end point are required to confirm our findings.

Our study showed that the greater risk of stroke recurrence with antidepressants in patients with diabetes. Diabetes patients with antidepressants had poor adherence with medication [27], which was associated with adverse outcomes such as higher glycosylated hemoglobin, systolic and diastolic blood pressure, and low-density lipoprotein cholesterol levels [28]. The risk of stroke recurrence associated with antidepressants use did not change materially in the analysis stratified by depression statuses. The indication for antidepressants was not only for the treatment of mood disorders but also for patients with various anxiety disorders and off-label use such as sleep disorder, chronic pain [29]. Although the increased risk of recurrent stroke with antidepressants use in prevalent users was lower but non-significant than in new users, the reduction in the risk with long-term drug users might happened [30] with the attrition for susceptible people [31]. We did not observe dose–response relationship between antidepressants use and stroke recurrence.

The strengths of our study included a large population-based follow-up study and the integrated details of prescription records such as the drug used, dosages, days of supply dispensed from database. However, our study had several potential limitations. First, the history of stroke prior to 1996 was unknown, but patients with any record of stroke before 1999 were excluded to reducing misclassified as an incident case. Second, the validity of diagnosis of stroke and the recording of prescription in database may influence our results, even the accuracy of recording stroke diagnoses and prescriptions in NHIRD was high [32]. Third, the data of antidepressants use in admission were excluded due to no information of the duration of use for each prescription from inpatient records. We used the length of stay for each admission as an estimate for duration of antidepressants exposure, and we found the association weakened but remained statistically significant. Fourth, we not only lacked for lifestyle information such as weight, drinking, or smoking status, but also for any data on stroke location or severity. Fifth, we were unable to assess the adherence of the antidepressants because the information is not available in the claims database. Poor adherence to antidepressant treatment may lead to misclassification of exposures resulting in under-estimation of the drug effect. Sixth, misclassification of depression might occur if a patient did not seek medical care for his or her depressive disorders or if diagnostic codes were accurate. In our analysis, depression was treated as a confounder in multivariable analysis. Misclassification of depression may result in residual confounding. Finally, we cannot draw conclusions on causal association from our analysis, as this is an observational study. This study was national representative and population-based. The results can be generalizable to populations in Chinese ethnicity.

## Conclusions

We found that use of antidepressants was associated with an increased risk of stroke recurrence, especially for ischemic stroke. TCAs, SSRIs, the group of others, or multiple types users had increased risk of stoke recurrence. In addition, a higher risk associated with antidepressants was observed in patients with diabetes. Although those patients had low dosage of antidepressants, the closely monitoring the side effects was necessary, particularly for diabetes patients.

## References

[1] World Health Organization: The Global Burden of Disease: 2004 Update.

[2] Mukherjee D, Patil CG (2011). Epidemiology and the global burden of stroke. World Neurosurg.

[3] Tseng MC, Lin HJ (2009). Readmission after hospitalization for stroke in Taiwan: results from a national sample. J Neurol Sci.

[4] Samsa GP, Bian J, Lipscomb J, Matchar DB (1999). Epidemiology of recurrent cerebral infarction: a medicare claims-based comparison of first and recurrent strokes on 2-year survival and cost. Stroke.

[5] Chen Y, Patel NC, Guo JJ, Zhan S (2007). Antidepressant prophylaxis for poststroke depression: a meta-analysis. Int Clin Psychopharmacol.

[6] Chollet F, Tardy J, Albucher JF, Thalamas C, Berard E, Lamy C, et al. Fluoxetine for motor recovery after acute ischaemic stroke (FLAME): a randomised placebo-controlled trial. Lancet Neurol. 2011;10:123–30. 10.1016/S1474-4422(10)70314-8 21216670

[7] Mikami K, Jorge RE, Adams HP, Davis PH, Leira EC, Jang M, et al. Effect of antidepressants on the course of disability following stroke. Am J Geriatr Psychiatr. 2011;19:1007–15. 10.1097/JGP.0b013e31821181b0 PMC3565535 21358384

[8] Hackett ML, Anderson CS, House AO, Xia J (2009). Interventions for treating depression after stroke. Stroke.

[9] Mead GE, Hsieh CF, Lee R, Kutlubaev MA, Claxton A, Hankey GJ, et al. Selective serotonin reuptake inhibitors (SSRIs) for stroke recovery. Cochrane Database Syst Rev. 2012;11:CD009286. 10.1002/14651858.CD009286.pub2 PMC6465036 23152272

[10] Wu CS, Wang SC, Cheng YC, Gau SS (2011). Association of cerebrovascular events with antidepressant use: a case-crossover study. Am J Psychiatry.

[11] Yuan HW, Wang CX, Zhang N, Bai Y, Shi YZ, Zhou Y, et al. Poststroke depression and risk of recurrent stroke at 1 year in a Chinese cohort study. PLoS One. 2012;7:e46906. 10.1371/journal.pone.0046906 PMC3474769 23082134

[12] Mortensen JK, Larsson H, Johnsen SP, Andersen G (2013). Post stroke use of selective serotonin reuptake inhibitors and clinical outcome among patients with ischemic stroke: a nationwide propensity score-matched follow-up study. Stroke.

[13] Pan A, Sun Q, Okereke OI, Rexrode KM, Hu FB (2011). Depression and risk of stroke morbidity and mortality: a meta-analysis and systematic review. JAMA.

[14] Pequignot R, Tzourio C, Peres K, Ancellin ML, Perier MC, Ducimetière P, et al. Depressive symptoms, antidepressants and disability and future coronary heart disease and stroke events in older adults: the Three City Study. Eur J Epidemiol. 2013;28:249–56. 10.1007/s10654-013-9765-3 23338904

[15] WHOCC ATC/DDD Index. http://www.whocc.no/atc_ddd_index/.

[16] Simon R, Makuch RW (1984). A non-parametric graphical representation of the relationship between survival and the occurrence of an event: application to responder versus non-responder bias. Stat Med.

[17] Schultz LR, Peterson EL, Breslau N (2002). Graphing survival curve estimates for time-dependent covariates. Int J Methods Psychiatr Res.

[18] Muhonen MG, Robertson SC, Gerdes JS, Loftus CM (1997). Effects of serotonin on cerebral circulation after middle cerebral artery occlusion. J Neurosurg.

[19] Razzaque Z, Heald MA, Pickard JD, Maskell L, Beer MS, Hill RG, et al. Vasoconstriction in human isolated middle meningeal arteries: determining the contribution of 5-HT1B- and 5-HT1F-receptor activation. Br J Clin Pharmacol. 1999;47:75–82. 10.1046/j.1365-2125.1999.00851.x PMC2014192 10073743

[20] Ramasubbu R (2004). Cerebrovascular effects of selective serotonin reuptake inhibitors: a systematic review. J Clin Psychiatr.

[21] Schlienger RG, Meier CR (2003). Effect of selective serotonin reuptake inhibitors on platelet activation: can they prevent acute myocardial infarction?. Am J Cardiovasc Drugs.

[22] Maurer-Spurej E, Pittendreigh C, Solomons K (2004). The influence of selective serotonin reuptake inhibitors on human platelet serotonin. Thromb Haemost.

[23] Hackam DG, Mrkobrada M (2012). Selective serotonin reuptake inhibitors and brain hemorrhage A meta-analysis. Neurology.

[24] Kharofa J, Sekar P, Haverbusch M, Moomaw C, Flaherty M, Kissela B, et al. Selective serotonin reuptake inhibitors and risk of hemorrhagic stroke. Stroke. 2007;38:3049–51. 10.1161/STROKEAHA.107.491472 17901378

[25] Bak S, Tsiropoulos I, Kjaersgaard JO, Andersen M, Mellerup E (2002). Selective serotonin reuptake inhibitors and the risk of stroke: a population-based case–control study. Stroke.

[26] Douglas I, Smeeth L, Irvine D (2011). The use of antidepressants and the risk of haemorrhagic stroke: a nested case control study. Br J Clin Pharmacol.

[27] Caughey GE, Preiss AK, Vitry AI, Gilbert AL, Ryan P, Shakib S, et al. Does antidepressant medication use affect persistence with diabetes medicines? Pharmacoepidemiol Drug Saf. 2013;22:615–22. 10.1002/pds.3424 23447430

[28] Ho PM, Rumsfeld JS, Masoudi FA, McClure DL, Plomondon ME, Steiner JF, et al. Effect of medication nonadherence on hospitalization and mortality among patients with diabetes mellitus. Arch Intern Med. 2006;166:1836–41. 10.1001/archinte.166.17.1836 17000939

[29] Wu CS, Shau WY, Chan HY, Lee YC, Lai YJ, Lai MS. Utilization of antidepressants in Taiwan: a nationwide population-based survey from 2000 to 2009. Pharmacoepidemiol Drug Saf. 2012;21:980–8. 10.1002/pds.3255 22511574

[30] de Abajo FJ, Gil MJ, Bryant V, Timoner J, Oliva B, García-Rodríguez LA. Upper gastrointestinal bleeding associated with NSAIDs, other drugs and interactions: a nested case–control study in a new general practice database. Eur J Clin Pharmacol. 2013;69:691–701. 10.1007/s00228-012-1386-3 22955795

[31] Moride Y, Abenhaim L (1994). Evidence of the depletion of susceptibles effect in non-experimental pharmacoepidemiologic research. J Clin Epidemiol.

[32] Cheng CL, Kao YH, Lin SJ, Lee CH, Lai ML (2011). Validation of the National Health Insurance Research Database with ischemic stroke cases in Taiwan. Pharmacoepidemiol Drug Saf.

